# Evaluation of a novel particle-based multi-analyte technology for the detection of anti-fibrillarin antibodies

**DOI:** 10.1007/s12026-021-09197-1

**Published:** 2021-04-28

**Authors:** Michael Mahler, Grace Kim, Fabrece Roup, Chelsea Bentow, Nicole Fabien, David Goncalves, Boaz Palterer, Marvin J. Fritzler, Danilo Villalta

**Affiliations:** 1 Research and Development, Inova Diagnostics, San Diego, CA 92131 USA; 2 Immunology Department, Lyon-Sud Hospital, Hospices Civils de Lyon, Claude Bernard, Pierre-Benite, France; 3 University Lyon I, University of Lyon, Pierre-Benite, France; 4 Department of Clinical and Experimental Medicine, Unit of Allergology and Clinical Immunology, University of Florence, Florence, Italy; 5 Department of Medicine, Cumming School of Medicine, University of Calgary, Calgary, AB T2N4N1 Canada; 6 Immunologia E Allergologia, Ospedale S. Maria degli Angeli, Pordenone, Italy

**Keywords:** Systemic sclerosis, Autoantibodies, Multi-analyte, Immunoassay

## Abstract

Systemic sclerosis (SSc) is a heterogeneous autoimmune disease associated with several anti-nuclear antibodies (ANA), including those in the classification criteria (anti-centromere, anti-topoisomerase I (Scl-70), anti-RNA Pol III). However, the presence of less common antibodies such as anti-fibrillarin (U3-RNP) that generate a clumpy nucleolar pattern by HEp-2 indirect immunofluorescence assay (IFA, ICAP AC-9) are considered disease specific and are with clinical subsets of SSc, therefore playing a role in diagnosis and prognosis. A specific and sensitive anti-fibrillarin assay would be an important addition to serological diagnosis and evaluation of SSc. The goal of this study was to evaluate a new particle-based multi-analyte technology (PMAT) for the measurement of anti-fibrillarin antibodies. A total of 149 patient samples were collected including 47 samples from France (Lyon and Paris, *n* = 32) and Italy (Careggi Hospital, Florence, *n* = 15) selected based on AC-9 HEp-2 IFA staining (> 1:640, clumpy nucleolar pattern) and 102 non-SSc controls (inflammatory bowel disease (IBD) *n* = 20, Sjögren’s syndrome (SjS) *n* = 20, infectious disease (ID) *n* = 7, systemic lupus erythematosus (SLE) *n* = 17, rheumatoid arthritis (RA) *n* = 17, and healthy individuals (HI) *n* = 21). All samples were tested on the anti-fibrillarin PMAT assay (research use only, Inova Diagnostics, USA). Additionally, the 47 anti-fibrillarin positive samples were also tested on PMAT assays for detecting other autoantibodies in ANA-associated rheumatic diseases (AARD). Anti-fibrillarin antibody data performed by fluorescence enzyme immunoassay (FEIA, Thermo Fisher, Germany) was available for 34 samples. The anti-fibrillarin PMAT assay was positive in 31/32 (96.9%, France) and 12/15 (80.0%, Italy) of samples preselected based on the AC-9 IIF pattern (difference *p* = 0.09). Collectively, the PMAT assay showed 91.5% (95% confidence interval (CI): 80.1–96.6%) sensitivity with 100.0% (95% CI: 96.4–100.0%) specificity in non-SSc controls. Strong agreement was found between PMAT and FEIA with 100.0% positive qualitative agreement (34/34) and quantitative agreement (Spearman’s rho = 0.89, 95% CI: 0.77.9–0.95%, *p* < 0.0001). Although most anti-fibrillarin positive samples were mono-specific (69.8%), some expressed additional antibodies (namely Scl-70, centromere, dsDNA, Ro52, Ro60, SS-B, Ribo-P, DFS70, and EJ). In conclusion, this first study on anti-fibrillarin antibodies measured using a novel PMAT assay shows promising results where the new PMAT assay had high level of agreement to FEIA for the detection of anti-fibrillarin antibodies. The availability of novel AFA assays such as PMAT might facilitate the clinical deployment, additional studies, standardization efforts, and potentially consideration of AFA for next generations of the classification criteria.

**Supplementary Information:**

The online version contains supplementary material available at 10.1007/s12026-021-09197-1.

## Introduction

Systemic sclerosis (SSc) is a heterogeneous autoimmune disease associated with several clinical features: fibrosis of the skin and internal organs, dysregulation of the immune system, and vasculopathy [1]. Mortality increases with internal organ involvement, especially the heart and kidney, as well as interstitial lung disease (ILD) and pulmonary arterial hypertension (PAH) [2]. Historically, the patterns of skin fibrosis were used to determine the disease severity and classify the patients into two subsets: diffuse cutaneous SSc (dcSSc) and limited cutaneous SSc (lcSSc) [3, 4]. Clinical features of dcSSc typically include skin thickening that extends proximally above the elbows and knees and on the chest while lcSSc typically exhibits skin thickening restricted to the face and distal upper extremities [5, 6]. As an adjunct to a clinical diagnosis, laboratory testing, particularly using autoantibodies, is used to aid in diagnosis and prognosis [7].

The presence of anti-nuclear antibodies (ANA) including those in the classification criteria (anti-centromere, anti-topoisomerase I (Scl-70), anti-RNA Pol III), and others such as ribonuclear proteins (anti-U11/U12, anti-U1 RNPC, anti-U3 RNP), and nucleolar antigens (anti-Th/To, anti-Ku, and anti-PM/Scl) have been utilized in research and diagnostic assays [6, 8–10]. While assays for the detection of the major SSc-related antibodies (anti-Scl-70, anti-centromere, and anti-RNA Pol III) are widely available, the detection of non-criteria antibodies is largely limited to immunoprecipitation (IP) and line immune assays (LIA) or dot blots. Validation and standardization of the immunoassays to detect these antibodies is still in progress and there are still significant differences between assays even for established autoantibody markers [11, 12].

Anti-fibrillarin (U3-RNP) is an example of an autoantibody that needs standardization. In ANA HEp-2 IIF, anti-fibrillarin autoantibodies are characterized by an irregular, clumpy staining of the nucleoli and with reticular mitosis at the metaphase and telophase plates (International Consensus on ANA Patterns (ICAP), AC-9) [13, 14]. However, the distinction between subtypes of nucleolar patterns is difficult even between highly trained experts and, therefore, the confirmation by antigen-specific immunoassays is recommended [10]. Historically, anti-fibrillarin antibodies were difficult to detect by western immunoblotting; hence, early studies relied on IP, including IP of radiolabeled fibrillarin produced by in vitro transcription and translation assays [15]. LIAs based on recombinant fibrillarin have been shown to be a rapid and less complex method with excellent agreement but well-known lower sensitivity than IP methods and the high specificity and more sensitive method of fluorescence enzyme immunoassay (FEIA) [16, 17].

Clinically, anti-fibrillarin autoantibodies have been associated with dcSSc, increased incidence of pulmonary arterial hypertension, skeletal muscle disease, severe cardiac involvement, and gastrointestinal dysmotility [18]. They also have been associated with certain HLA-DQB1 alleles, specific ethnicities, such as Afro-Caribbean origin, identify younger SSc patients, and display a higher prevalence of myositis [14, 15, 19, 20]. It has also been reported that anti-fibrillarin antibodies were associated with native American ethnicity and were mortality independent predictors in those affected with SSc [21].

At present, the assays highlighted above are convenient tools for the detection of anti-fibrillarin antibodies and other SSc antibodies, but without strong clinical evidence and a standardized platform, additional work is important [22–24]. Anti-fibrillarin antibodies aid in the prediction of mortality, improve diagnostic accuracy, and can be useful in early diagnosis since autoantibodies are present in very early SSc before clinical symptoms appear [25–27]. This study aimed to evaluate a new particle-based multi-analyte technology (PMAT) for the measurement of anti-fibrillarin antibodies.

## Materials and methods

### Patient characteristics and samples

**Fig. 1:**
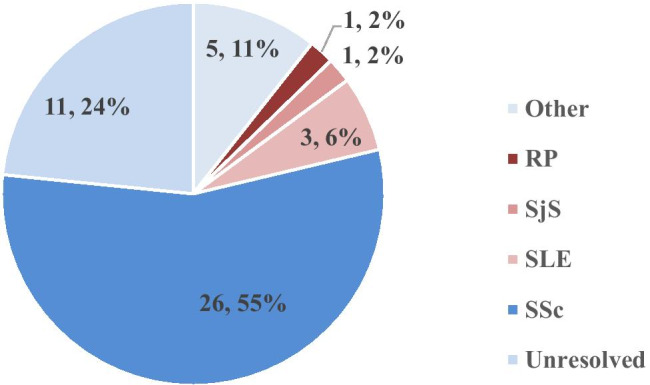
Summary of patient diagnosis for 47 samples collected by nucleolar pattern on indirect immunofluorescence (IIF) using HEp-2 cells with suspected anti-fibrillarin antibodies. The most prevalent diagnosis was systemic sclerosis (SSc, *n* = 26, 55%). Other diseases included systemic lupus erythematosus (SLE, *n* = 3, 6%), Raynaud’s phenomenon (RP, *n* = 1, 2%), and Sjögren’s syndrome (SjS, *n* = 3, 2%). Eleven of the patients remain unresolved without clinical diagnosis (24%) and 5 patients (11%) made up other diseases (Crohn’s disease, *n* = 2, Devic’s disease, *n* = 1, and cancer, *n* = 2)

A total of 149 serum samples were collected to evaluate the PMAT assay. Forty-seven samples suspected to contain anti-fibrillarin antibodies based on staining pattern by IIF (titer > 1:640, clumpy nucleolar pattern, and reticular mitosis) were collected in France (two sites, *n* = 32), and in Italy (Careggi Hospital, *n* = 15). Of the samples collected with typical nucleolar pattern in initial screening, most patients resulted with a diagnosis of SSc and other confirmed diseases, while a portion of patients remain with unresolved diagnosis (Fig. 1). To assess specificity, patient samples from other autoimmune and non-autoimmune disorders were included in the study as controls (inflammatory bowel disease (IBD) *n* = 20, Sjögren’s syndrome (SjS) *n* = 20, infectious disease (ID) *n* = 7, systemic lupus erythematosus (SLE) *n* = 17, rheumatoid arthritis (RA) *n* = 17, and healthy individuals (HI) *n* = 21). The study was conducted in accordance with the Declaration of Helsinki Ethical Principles and Good Clinical Practices and was approved by an independent local ethics committee.

### ANA reference panel

The anti-nuclear antibody (ANA) reference panel from the Center of Disease Control (CDC) comprises 12 ANA reference samples with different characterized ANA reactivity and was tested to evaluate the specificity of the fibrillarin PMAT assay (Table 1). Reference sera was originally collected for standardizing fluorescence ANA and for establishing antibodies to established specificity [28]. ANA human reference serum #6 is characterized as nucleolar pattern (ICAP AC-9) and anti-fibrillarin (U3 RNP) which is expected to be positive on the PMAT assay to confirm the presence of anti-fibrillarin antibodies.

**Table 1:** Established autoantibody profiles of anti-nuclear antibody (ANA) reference panel along with results for Aptiva fibrillarin assay on the particle-based multi-analyte technology (PMAT) measured in median fluorescent intensity (MFI) units

Sample no	ANA human reference serum	Fluorescent anti-nuclear antibody pattern on HEp-2 cells and/or antibody content	Aptiva fibrillarin PMAT result (Interpretation, MFI)
1	ANA human reference serum #01	Fluorescence ANA (homogeneous/rim pattern); anti-native DNA dsDNA	Negative (48)
2	ANA human reference serum #02	Fluorescence ANA (speckled pattern); anti-SS-B/La	Negative (50)
3	ANA human reference serum #03	Fluorescence ANA (speckled pattern)	Negative (62)
4	ANA human reference serum #04	Anti-U1 RNP (nuclear RNP)	Negative (54)
5	ANA human reference serum #05	Anti-Sm	Negative (83)
6	ANA human reference serum #06	Nucleolar pattern; anti-fibrillarin (U3 RNP)	Positive (4819)
7	ANA human reference serum #07	Anti-SS-A/Ro	Negative (45)
8	ANA human reference serum #08	Centromere pattern	Negative (56)
9	ANA human reference serum #09	Anti-Scl-70 (DNA topoisomerase I)	Negative (62)
10	ANA human reference serum #10	Anti-Jo1 (histidyl-tRNA synthetase)	Negative (48)
11	ANA human reference serum #11	Anti-PM/Scl	Negative (56)
12	ANA human reference serum #12	Anti-ribosomal P	Negative (46)

### Immunoassays

#### Aptiva®—particle-based multi-analyte technology

All patient samples were tested using the Aptiva® anti-Fibrillarin reagents on the Aptiva® instrument (research use only, Inova Diagnostics, San Diego, USA). To complete the full antibody profile, the 47 samples collected for anti-fibrillarin testing were additionally tested on PMAT assays for detecting other autoantibodies in connective tissue disease (research use only, Aptiva CTD essential reagent: dsDNA, centromere, Scl-70, RNP, Sm, Ro60, Ro52, SS-B, Ribo-P, Jo-1, DFS70, Aptiva CTD comprehensive reagent: RNA Pol III, Th/To (Rpp25), Th/To (Rpp38), PM/Scl, Ku, BICD2, PCNA, and Aptiva myopathy reagent: Jo-1, PL-7, PL-12, EJ, MDA-5, NXP-2, TIF1y, Mi-2, SAE-1, HMGCR, SRP, Inova Diagnostics, San Diego, USA). The PMAT technology has been previously described [29]. Aptiva® reagents come in a cartridge format and are designed solely for use with the Aptiva® instrument, a fully automated random-access benchtop system. Aptiva utilizes laser-induced fluorescence for detection and simultaneously measures multiple antibodies within a small serum sample (5–10 μL). The Aptiva® instrument is equipped with a high-resolution digital camera, as well as all the hardware, liquid handling, and user interface software necessary to perform the assays.

The Aptiva® anti-fibrillarin PMAT reagents are solid-phase immunoassays for the semi-quantitative determination of anti-fibrillarin antibodies in human serum. Antibody isotype is determined using an anti-human IgG reagent. A full-length human recombinant fibrillarin antigen is covalently bound to uniquely identifiable paramagnetic microparticles. Prior to use in the Aptiva® instrument, the reagent cartridge, containing all required components, is prepared by piercing the sealed reagent tubes with the cartridge lid. Once placed onboard, the Aptiva® instrument automatically rehydrates the microparticles. A patient serum sample is pre-diluted by the Aptiva® instrument with sample buffer in a small disposable plastic cuvette. Small amounts of the diluted patient serum, the microparticles, and the assay buffer are all combined into a second cuvette, mixed, and then incubated for 9.5 min at 37 °C. The magnetized microparticles are washed several times followed by the addition of phycoerythrin (PE)-conjugated anti-human IgG antibody, and again incubated for 9.5 min at 37 °C. The magnetized microparticles are washed repeatedly, before being transferred to the optical module for quantitation.

Multiple digital images are generated by the Aptiva® system to identify and count the unique analyte microparticles, as well as determine the amount of PE conjugate bound to each particle. The PE fluorescence is measured as the median fluorescent intensity (MFI) for each analyte and is proportional to the amount of conjugated anti-human IgG antibody bound to human immunoglobulin bound to the antigen on the microparticle. The system converts the measured MFI into units using analyte-specific calibrated 4-parameter logistic (4PL) curves.

#### Fluorescence enzyme immunoassay

For method comparison between anti-fibrillarin assays, 34 samples were tested by a fluorescence enzyme immunoassay (FEIA, EliA® fibrillarin, Thermo Fisher, Germany) in the routine diagnostic labs. The assay was performed according to the manufacturer’s instructions and thresholds defined (< 7 U/mL negative, 7–10 U/mL equivocal, > 10 U/mL positive). In short, the EliA fibrillarin FEIA contains human recombinant fibrillarin protein bound to wells which is incubated with patient sera, then after washing incubates with a fluorochrome-conjugated anti-human IgG antibody and the fluorescence determined in units/mL is proportional to the amount of antibody present in patient sera.

#### Indirect immunofluorescence on HEp-2 cells

For characterization of samples in routine (some with diagnosis and others undetermined), samples were screened using indirect immunofluorescence (IIF) on HEp-2 cells to identify the clumpy nucleolar pattern and reticular mitosis [18].

#### Line immunoassay and western blotting technique

Samples collected from the University of Lyon had previous testing performed on LIA-DB (line immunoassay, dot blot, Dtek, Belgium) for 5 samples where available [14]. In addition, western blotting to detect anti-fibrillarin antibodies was performed using rat liver as previously described for 8 samples [14] (Supplementary Table 1).

### Analytical performance studies of Aptiva fibrillarin PMAT method

#### Precision

Three serum samples derived from 3 unique samples containing anti-fibrillarin antibodies were selected. Aliquots of each sample were created to be used for a minimum of 3 days of testing, with additional aliquots being made in case of additional testing being required. Aliquots were stored in sealed vials at 2–8 °C until and in between testing. The precision study followed the 3-day, 1 run per day, 3 replicates per run design (3 × 1 × 3). All samples were run on the same instrument, using one reagent lot. New sample aliquots were used upon a repeated run. The acceptance criteria for the precision study were defined as the total %CV for each sample must be ≤ 12%.

#### Linearity

For the linearity study, analytical measuring range (AMR) was defined using two (*n* = 2) high titer positive samples tested in twofold serial dilutions, at least in duplicate, covering the visual range of the assay (from high to low plateaus). These results were then input into a “theoretical” master curve in the Aptiva system software to generate a 4PL curve. The acceptance criteria were defined as determining the AMR range, where sequential unit values do not change more than 20%. Duplicates had < 10% CV to be acceptable.

### Statistical analysis

All statistical analyses were performed by Analyse-it® for Excel method evaluation software (version 5.01; Leeds, UK). Diagnostic sensitivity and specificity of all assays were calculated and compared. Diagnostic efficacy was assessed by receiver operating characteristic (ROC) analysis. Spearman’s correlation was carried out to analyze concordance between methods. If needed, the Haldane-Anscombe correction [30, 31] was used to recalculate likelihood ratios (LR), odds ratios (OR), and limits resulting to infinity values. A preliminary cut-off for the PMAT assay was defined based on internal clinical validation/evaluations performed by the manufacturer.

## Results

**Fig. 2:**
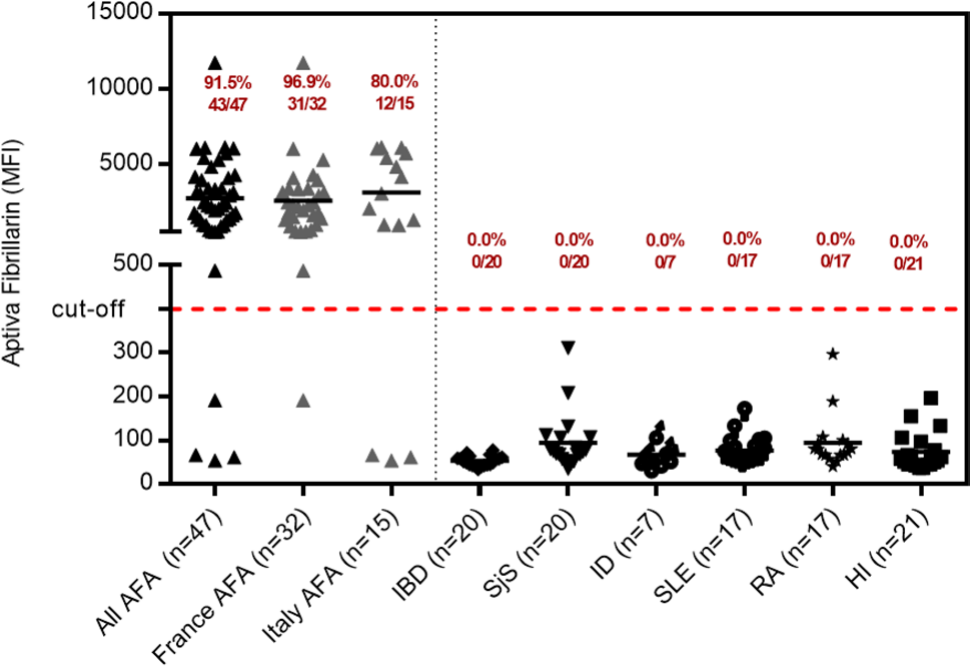
Anti-fibrillarin antibody levels measured using the particle-based multi-analyte technology (PMAT) assay. Anti-fibrillarin antibodies were significantly higher in the group of samples selected based on the suspicion of anti-fibrillarin reactivity. AFA = anti-fibrillarin antibodies, SjS = Sjögren’s syndrome, RA = rheumatoid arthritis, IBD = inflammatory bowel disease, ID = infectious disease, HI = healthy individuals, MFI = median fluorescent intensity

**Fig. 3:**
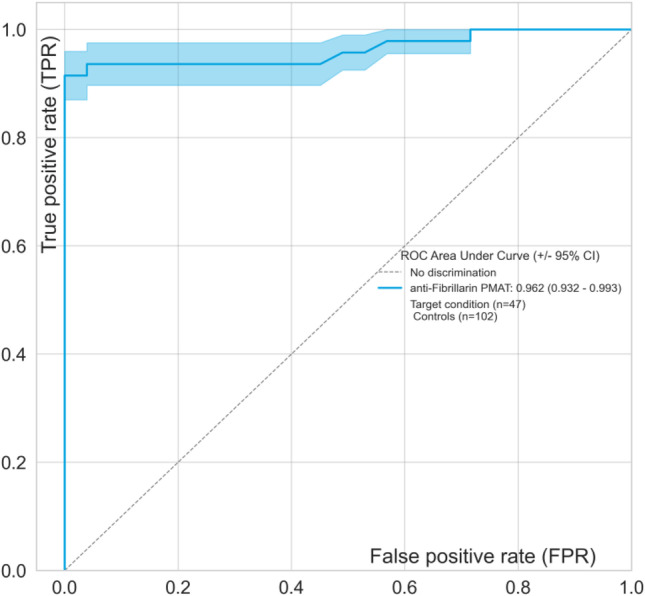
Receiver operating characteristic (ROC) curve analysis for anti-fibrillarin antibodies. The ROC analyzes the ability of the Aptiva fibrillarin particle-based multi-analyte technology (PMAT) assay to discriminate samples suspected to have anti-fibrillarin antibodies (*n* = 47) versus controls (*n* = 102). The area under the ROC curve (AUC) is listed in parentheses in the graph

**Fig. 4:**
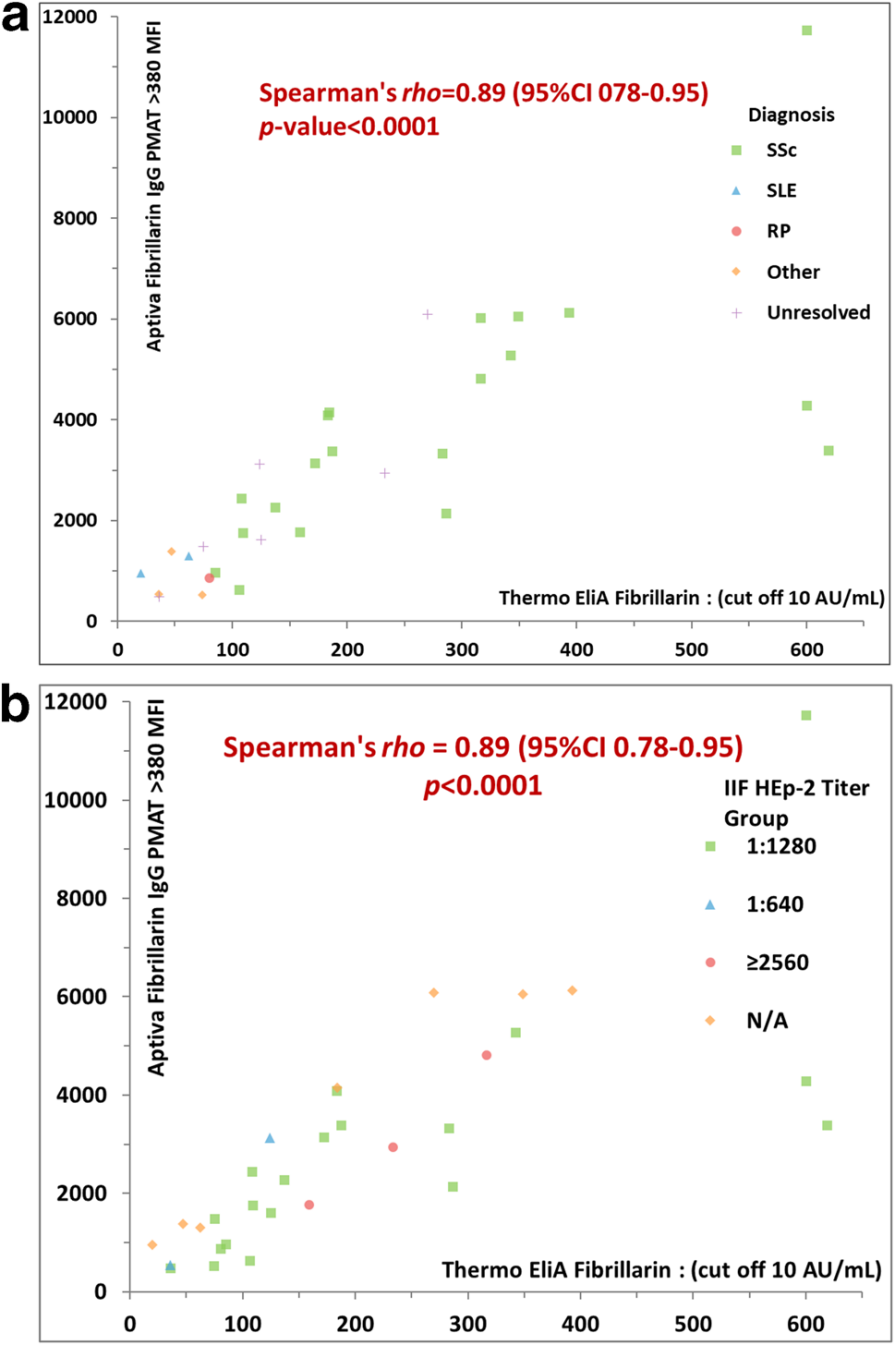
Spearman’s quantitative correlations between comparing two assays for the detection of anti-fibrillarin antibodies. Aptiva fibrillarin particle-based multi-analyte technology (PMAT) assay versus the fluorescence enzyme immunoassay (FEIA) using 32 patient samples where semi-quantitative results for FEIA were available. A high level of agreement was found between PMAT and FEIA (rho = 0.89). **a** The correlation with data points coded based on patient groups (diagnosis available or unresolved). **b** The same data coded by indirect immunofluorescence titer

**Fig. 5:**
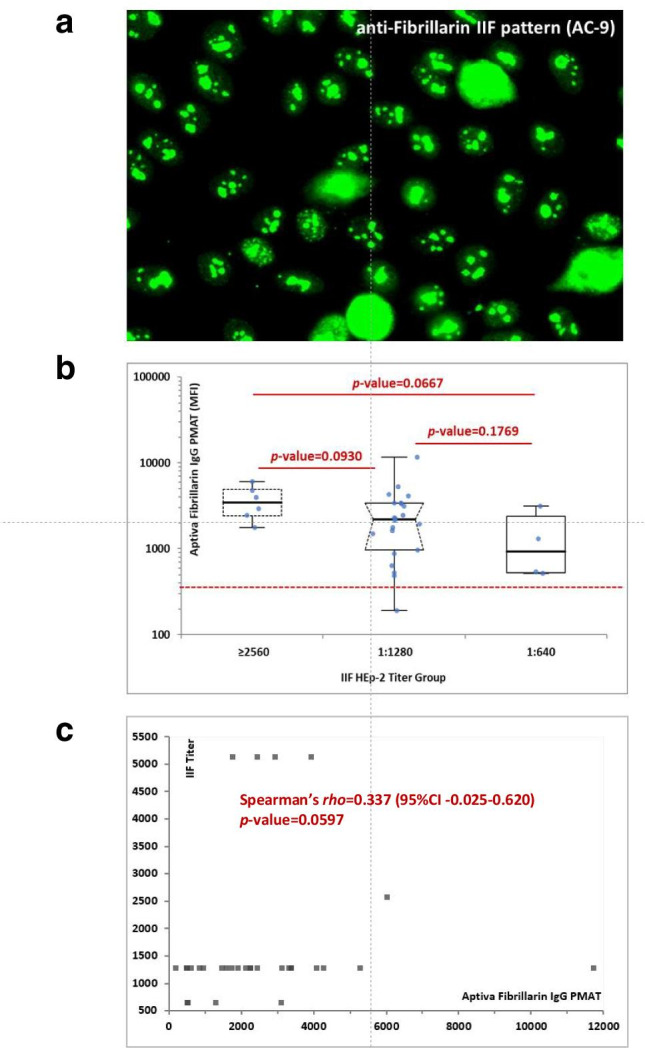
Association of anti-fibrillarin antibodies and indirect immunofluorescence (IIF). **a** Representative clumpy nucleolar staining pattern by IIF on HEp-2 cells which corresponds to international consensus on ANA patterns (ICAP) AC-9. **b** Anti-fibrillarin antibody levels on the particle-based multi-analyte technology (PMAT) assay expressed in median fluorescent intensity (MFI) units compared to titer of indirect immunofluorescence (IIF) on HEp-2 cells with AC-9 clumpy nucleolar pattern. **c** Spearman’s analysis of anti-fibrillarin antibody levels on PMAT compared to IIF titer showed a trend of correlation but not significant (*p* = 0.0597)

**Fig. 6:**
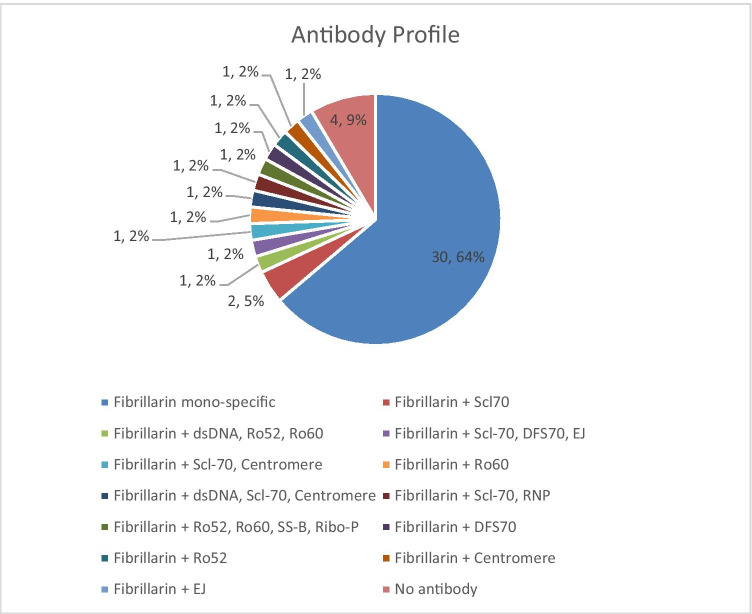
Overlap of anti-fibrillarin with other autoantibodies in the cohort measured with the particle-based multi-analyte technology (PMAT) system. Overlap between anti-fibrillarin positivity and other antibodies is found in 30.2% of cases, whereas 64.0% of patient samples were mono-specific for anti-fibrillarin. Other antibody positivity included anti-Scl-70, anti-centromere, anti-dsDNA, anti-Ro52, anti-Ro60, anti-SS-B, anti-Ribo-P, anti-DFS70, and anti-EJ antibodies

The anti-fibrillarin PMAT assay showed positivity in 31/32 (96.9%, France) and in 12/15 (80.0%, Italy) of samples preselected based on HEp-2 IIF pattern (Fig. 2), respectively. Both, the prevalence (*p* = 0.09) and the antibody levels (*p* = 0.5081) were not statistically different between the two countries. Collectively, the PMAT assay showed 91.5% (95% confidence interval (CI): 80.1–96.6%) sensitivity with 100.0% (95% CI: 96.4–100.0%) specificity in the controls using a preliminary cut-off of 380 MFI defined by the specificity of this testing (Table 2, Fig. 3). When analyzing the specificity for SSc in the nucleolar samples alone, a higher threshold of 1612 MFI resulted in 22/26 (84.6%) positive in SSc versus 7/21 (33.3%) in the other samples not confirmed with SSc; however, some patient diagnoses remain unresolved and have high levels of anti-fibrillarin antibodies. In addition, excellent agreement was found between PMAT and FEIA with 100.0% positive qualitative agreement (34/34) and good quantitative agreement (Spearman’s rho = 0.89, 95% CI: 0.77.9–0.95%, *p* < 0.0001, Fig. 4). For comparison to IIF titer, antibody levels on the PMAT assay were plotted against IIF titers as < 1:640, 1:1280, and ≥ 1:2560 with no statistical significance difference between IIF titers (*p* > 0.0667, *p* > 0.1769, *p* > 0.0.930, Fig. 5). For precision, the total %CV ranged between 3.5 and 5.7% for all samples. For the ANA reference panel, the anti-fibrillarin PMAT assay showed strong positive for ANA reference serum #6 (nucleolar pattern and anti-fibrillarin (U3 RNP)), while being negative on the other 11 samples. Testing of the nucleolar samples (*n* = 47) using PMAT assays for other ANA-associated rheumatic disease (AARD) antibodies demonstrated that most samples were mono-specific (69.8%, 30/43) (Supplementary Table 1). However, in 13/47 (27.7%) of the specimens, other antibodies were detected. Among those, 6/13 (46.2%) had SSc-associated antibodies (5 anti-Scl-70, 3 anti-centromere, Fig. 6). The four samples which showed nucleolar positive on IIF but fibrillarin PMAT assay negative remain undetermined for a specific antibody including those associated with a nucleolar pattern (Th/To, and PM/Scl). Interestingly, two patients expressed both anti-fibrillarin and anti-DFS70 antibodies, a combination that has not been reported before. For the linearity study, the AMR was identified as 128 MFI to 1265 MFI with a cut-off at 380 MFI which demonstrated a good linear range for the assay. All samples fell within the acceptable criteria of within 10% CV between duplicates.

**Table 2:** Performance characteristics of the PMAT assay for the detection of anti-fibrillarin antibodies

Parameter	Fibrillarin PMAT
Sensitivity (95% confidence interval)	91.5% (80.1–96.6%)
Specificity (95% confidence interval)	100.0% (96.4–100.0%)
Likelihood ratio +	+ ∞
Likelihood ratio −	0.09
Odds ratio	+ ∞
Area under the curve	0.96 (0.92–1.00)

## Discussion

Anti-nuclear antibodies are important biomarkers in the diagnosis and prognosis of SSc patients [9, 10, 32]. Besides the classification criteria autoantibodies anti-centromere, anti-Scl-70, and anti-RNA Pol III, several other antibodies have proven useful in the management of SSc patients [32]. Among those, anti-fibrillarin (U3-RNP) antibodies were first described by Okano in 1992 and then intensively studied by others [13, 14]. The importance of anti-fibrillarin antibodies was also recognized during the generation of a reference panel for anti-nuclear antibodies (ANA) by the Center of Disease Control and Prevention (CDC) which includes a serum containing anti-fibrillarin antibodies [33]. However, despite the broad knowledge, anti-fibrillarin antibodies are not routinely used in all geographic regions partly due to limited availability of commercial immunoassays with regulatory clearance (i.e., FDA-approved assay). The immunoassays in relatively wide use today can be separated into three main groups: (a) line immunoassays (LIA) and dot blots, (b) FEIA, and (c) immunoprecipitation. The lack of accepted reference panels and the restricted availability of the gold-standard IP has limited the clinical development of novel SSc antibody assays like anti-fibrillarin [11, 12]. In addition, the technical difficulty to develop anti-fibrillarin ELISA assays (or similar tests) has limited reliable assay development and the clinical use [34]. However, anti-fibrillarin can be used in the early identification of very early SSc patients particularly those with internal organ involvement [19, 35]. In addition, those antibodies represent a prognostic marker and increase the diagnostic rate in specific ethnicities, such as native North Americans [14, 19, 21]. The novel PMAT assay for the detection of anti-fibrillarin antibodies represents the first particle-based liquid phase assay and expands the options for the detection of anti-fibrillarin antibodies, offering a fast, quantitative, and random-access assay. In this cohort, the PMAT assay showed high specificity (100.0%, Fig. 2, Table 2) in well-defined non-SSc controls (IBD, SjS, ID, SLE, RA, HI) while still maintaining good sensitivity in the nucleolar samples. When analyzing the specificity for SSc in the nucleolar samples alone, a higher cut-off was more specific to the confirmed SSc patients (84.6% versus 33.3% positive in the other samples not confirmed with SSc). However, most of the other patient diagnoses remain unresolved without confirmed diagnosis (11/21, 52.3%) and many had high levels of anti-fibrillarin antibodies which were found on both PMAT and FEIA (and by IIF). The new PMAT assay showed high agreement to FEIA in qualitative and quantitative analysis as well as with the AC-9 IIF pattern as defined by ICAP for anti-fibrillarin antibodies [18]. It is important to note that our samples were selected based on screening by IIF on HEp-2 cells followed by confirmation using LIA or FEIA which might have introduced a bias towards high titer samples. Therefore, it is conceivable that the agreement might be lower in unselected samples. However, IIF represents the recommended method for ANA testing. This study also confirmed that for the ANA reference panel, the anti-fibrillarin PMAT assay showed strong positive for the sample characterized as nucleolar pattern and anti-fibrillarin (U3 RNP), while demonstrating good specificity being negative on the other 11 samples. Profiling of the nucleolar samples (*n* = 47) by PMAT for AARD-associated antibodies demonstrated that most samples were mono-specific for anti-fibrillarin (69.8%, 30/43) which further supports the value of anti-fibrillarin testing in routine. Other antibodies were observed in 13/47 (27.7%) of the samples and a large portion had SSc-associated antibodies (6/13, 46.2%, Scl-70, centromere); however, the other antibodies found were related to other connective tissue diseases such as SLE (dsDNA, RNP, Ribo-P), and Sjögren’s syndrome (Ro/La). The findings of anti-fibrillarin reactivity in SLE and SjS patients in this cohort as well as the antibody profile suggest a potential overlap syndrome of the patient samples and can also be related to previous findings of anti-fibrillarin antibodies in patients with more severe disease or with lupus [36]. It would be interesting in future studies to explore the prevalence and clinical significance of nucleolar positive samples found in different CTD and determining the prevalence of those with anti-fibrillarin antibodies now that more reliable anti-fibrillarin assays are available [37]. It is also important to note that outside of fibrillarin, no other antibodies that show nucleolar pattern by HEp-2 IIF antibodies were found in any of the samples (RNA Pol III, Th/To, and PM/Scl); therefore, the four samples which showed nucleolar positive on IIF but fibrillarin PMAT assay negative remain undetermined for a specific antibody. Interestingly, two patients expressed both anti-fibrillarin and anti-DFS70 antibodies, a combination that has not been reported before and again reinforces the need for specific antibody testing in addition to IIF pattern identification. Further studies are warranted to investigate the clinical associations on larger SSc cohorts and the performance of the new method in combination with other clinical markers.

The prevalence of anti-fibrillarin antibodies in SSc has widely varied in previous studies (0.5–5.0%) especially among different geographic regions and differing patient populations [10]. A recent study using LIA found 1.3% of SSc patients positive for anti-fibrillarin antibodies in a Greek cohort of 158 patients [38]. Another large study by Otero et al. that included 1506 SSc patients tested by the same LIA confirmed the association of anti-fibrillarin antibodies with severe SSc and increased mortality [21]. In contrast, in 2018 Boonstra used the FEIA AFA for a study on 407 Dutch SSc patients and reported a prevalence of 4% [39]. Another European study described associations with younger age at disease onset (*p* = 0.02), male gender (*p* = 0.02), Afro-Caribbean descent (*p* < 0.001), Rodnan skin score (*p* = 0.01), and myositis (*p* = 0.01) using ELIA for the detection of anti-fibrillarin antibodies [14]. In a large study on 1000 American SSc patients and 50 healthy controls, a very high correlation between IP and LIA was observed [17]. However, no disease controls were included in this study.

Anti-fibrillarin antibodies measured by LIA were also studied in a cohort of SSc patients (*n* = 505) from Australia of which 1.2% tested positive [40]. Villalta evaluated the LIA and reported 0.48% positive patients in 210 SSc patients from Italy [26]. While LIA use has been prolific to detect autoantibodies associated with SSc including anti-fibrillarin antibodies, the LIA platform does have limitations. It has been previously discussed that LIA lacks antigen-specific calibration and controls (as part of the commercial kit). Consequently, the burden is on the laboratory to ensure expected performance which requires the collection of well-characterized patient samples and complemented with controls [41, 42]. This is of high relevance since the temperature at which the assay is performed can impact the results as reported by Ronnelid et al. in 2009 [42]. The availability of novel AFA assays such as PMAT might facilitate the clinical deployment, additional studies, standardization efforts, and potentially consideration of AFA for next generations of the classification criteria.

Systemic sclerosis is a challenging disease, impacting quality of life and often having a high morbidity and mortality [1, 2]. There is an unmet need for specific treatments for SSc properly validated through high-quality clinical trials [43]. While there are trials that are currently underway to assess the efficacy of novel drugs and biologics, none of these studies stratifies the patients by their autoantibody status [44–46].

For our study, we used an approach to enrich for AFA positive samples using IIF on HEp-2 followed by confirmation with a solid-phase assay as inclusion criterion [41]. This might not only represent some limitations, but also allow to reduce confidence intervals of statistical comparisons. Based on the rarity of SSc and the low prevalence of AFA in SSc, our cohort of 47 patient samples would, based on the known prevalence (0.5–5%), require SSc cohorts of 940 and 9400 patient samples.

In conclusion, this first study on anti-fibrillarin antibodies measured using a novel PMAT assay shows promising results. Further studies on large SSc cohorts are required to establish the clinical sensitivity and specificity of the assay and to validate the clinical associations known for anti-fibrillarin antibodies.

## Supplementary Information

Below is the link to the electronic supplementary material.

Supplementary file1 (DOCX 64 KB)

## Data Availability

Data are available upon request.

## Abbreviations

SSc: Systemic sclerosis
PMAT: Particle-based multi-analyte technology
