# Correction to: Evaluation of a novel particle-based multi-analyte technology for the detection of anti-fibrillarin antibodies

**DOI:** 10.1007/s12026-021-09206-3

**Published:** 2021-06-12

**Authors:** Michael Mahler, Grace Kim, Fabrece Roup, Chelsea Bentow, Nicole Fabien, David Goncalves, Boaz Palterer, Marvin J. Fritzler, Danilo Villalta

**Affiliations:** 1 Research and Development, Inova Diagnostics, San Diego, CA 92131 USA; 2 Immunology Department, Lyon-Sud Hospital, Hospices Civils de Lyon, Claude Bernard, Pierre-Benite, France; 3 University Lyon I, University of Lyon, Pierre-Benite, France; 4 Department of Clinical and Experimental Medicine, Unit of Allergology and Clinical Immunology, University of Florence, Florence, Italy; 5 Department of Medicine, Cumming School of Medicine, University of Calgary, Calgary, AB T2N4N1 Canada; 6 Immunologia E Allergologia, Ospedale S. Maria degli Angeli, Pordenone, Italy

**Correction to: Immunologic Research**

**https://doi.org/10.1007/s12026-021-09197-1**

The article Evaluation of a novel particle-based multi-analyte technology for the detection of anti-fibrillarin antibodies, written by Michael Mahler, Grace Kim, Fabrece Roup, Chelsea Bentow, Nicole Fabien, David Goncalves, Boaz Palterer, Marvin J. Fritzler, and Danilo Villalta, was originally published Online First without Open Access. With the author(s)’ decision to opt for Open Choice, the copyright of the article changed on May 13, 2021, to © The Author(s) 2021 and the article is forthwith distributed under a Creative Commons Attribution 4.0 International License, which permits use, sharing, adaptation, distribution, and reproduction in any medium or format, as long as you give appropriate credit to the original author(s) and the source, provide a link to the Creative Commons license, and indicate if changes were made. The images or other third party material in this article are included in the article’s Creative Commons license, unless indicated otherwise in a credit line to the material. If material is not included in the article’s Creative Commons license and your intended use is not permitted by statutory regulation or exceeds the permitted use, you will need to obtain permission directly from the copyright holder. To view a copy of this license, visit http://creativecommons.org/licenses/by/4.0.

The original article has been corrected.

