# Supplementary material for: Evaluation of a novel particle-based multi-analyte technology for the detection of anti-fibrillarin antibodies
Source: Immunol Res. 2021 Apr 28;69(3):239–48. doi: 10.1007/s12026-021-09197-1 (PMC8266783; doi:10.1007/s12026-021-09197-1)
Supplement: Supplementary file 1 — Supplementary file1 (DOCX 64 KB) [file 12026_2021_9197_MOESM1_ESM.docx]

## Supplementary

**Supplementary Table 1. Detailed summary of 47 samples collected with suspected anti-fibrillarin antibodies, including the overlap for other autoantibodies with anti-fibrillarin antibodies measured with the particle-based multi-analyte technology (PMAT) system.** Results from FEIA (EliA® Fibrillarin, Thermo Fisher, Germany), in house western blot as described by Tall, and LIA-DB (DTEK, Belgium) were from routine lab testing.

| ID | Clinical Diagnosis | Age | IIF HEp-2 titer | FEIA (AU/mL)  c/o = 10 | PMAT (MFI)  c/o = 380 | Western blot | LIA-DB | Other Antibodies (PMAT Result) |
| --- | --- | --- | --- | --- | --- | --- | --- | --- |
| 1 | SSc | 19 | 1:1280 | N/P | 191 | positive | N/P | None |
| 2 | SSc | 27 | 1:1280 | 619.0 | **3389** | positive | N/P | None |
| 3 | SSc | 35 | 1:1280 | 342.0 | **5284** | positive | N/P | None |
| 4 | SSc | 58 | 1:1280 | 137.0 | **2271** | positive | N/P | dsDNA, Ro52, Ro60 |
| 5 | SSc | 21 | 1:1280 | 600.0 | **4283** | N/P | N/P | None |
| 6 | Unresolved | 66 | 1:1280 | 36.0 | **488** | N/P | N/P | Scl-70, DFS70, EJ |
| 7 | SSc | 49 | 1:1280 | 183.0 | **4090** | N/P | N/P | None |
| 8 | SSc | 70 | 1:1280 | 187.0 | **3387** | N/P | N/P | dsDNA, Scl-70, Centromere |
| 9 | SSc | 29 | 1:1280 | 600.0 | **11731** | positive | positive +++ | None |
| 10 | SSc | 71 | 1:1280 | 106.0 | **636** | positive | N/P | None |
| 11 | Unresolved | 36 | 1:1280 | 125.0 | **1612** | positive | N/P | None |
| 12 | RP | 33 | 1:1280 | 80.0 | **871** | positive | N/P | None |
| 13 | SSc | 28 | 1:1280 | 286.0 | **2148** | N/P | N/P | None |
| 14 | Other | 12 | 1:1280 | 74.0 | **526** | N/P | N/P | None |
| 15 | SSc | 66 | 1:1280 | 85.0 | **967** | N/P | N/P | Ro60 |
| 16 | Unresolved | 31 | 1:1280 | 75.0 | **1488** | N/P | N/P | None |
| 17 | Other | 25 | N/P | 47.0 | **1386** | N/P | N/P | dsDNA, Scl-70, Centromere |
| 18 | SSc | 9 | 1:1280 | 172.0 | **3139** | N/P | N/P | None |
| 19 | Other | 14 | 1:1280 | N/P | **1930** | N/P | positive (scan 105) | None |
| 20 | SjS | 46 | 1:640 | N/P | **1307** | N/P | positive (scan 30) | None |
| 21 | SSc | 41 | 1:1280 | N/P | **2266** | N/P | positive (scan 100) | None |
| 22 | Unresolved | 54 | 1:640 | N/P | **523** | N/P | positive (scan 50) | None |
| 23 | Unresolved | N/P | N/P | N/P | 66 | N/P | N/P | None |
| 24 | Other | N/P | N/P | N/P | 54 | N/P | N/P | None |
| 25 | SSc | 31 | N/P | 184.0 | **4155** | N/P | N/P | None |
| 26 | SSc | 83 | N/P | 393.0 | **6130** | N/P | N/P | RNP, Scl-70 |
| 27 | SSc | 67 | N/P | 349.0 | **6048** | N/P | N/P | Scl-70 |
| 28 | SLE | 29 | N/P | 20.0 | **951** | N/P | N/P | None |
| 29 | SLE | 39 | N/P | 62.0 | **1298** | N/P | N/P | None |
| 30 | SSc | 32 | N/P | N/P | **5403** | N/P | N/P | None |
| 31 | Unresolved | N/P | N/P | 270.0 | **6090** | N/P | N/P | None |
| 32 | Unresolved | N/P | N/P | N/P | 61 | N/P | N/P | None |
| 33 | Unresolved | N/P | N/P | N/P | **2066** | N/P | N/P | None |
| 34 | Unresolved | N/P | N/P | N/P | **5729** | N/P | N/P | None |
| 35 | SSc | N/P | 1:5120 | 159 | **1773** | N/P | N/P | None |
| 36 | Unresolved | N/P | 1:5120 | 233 | **2942** | N/P | N/P | None |
| 37 | Unresolved | N/P | 1:640 | 124 | **3125** | N/P | N/P | None |
| 38 | SLE | N/P | 1:5120 | N/P | **3951** | N/P | N/P | Ro52, Ro60, SS-B, Ribo-P |
| 39 | SSc | N/P | 1:2560 | 316 | **6032** | N/P | N/P | None |
| 40 | SSc | N/P | 1:1280 | 109 | **1756** | N/P | N/P | Scl-70 |
| 41 | Other | N/P | 1:640 | 36 | **543** | N/P | N/P | DFS70 |
| 42 | SSc | N/P | 1:1280 | 283 | **3333** | N/P | N/P | Ro52 |
| 43 | SSc | N/P | 1:5120 | N/P | **2463** | N/P | N/P | None |
| 44 | SSc | N/P | 1:1280 | 108 | **2451** | N/P | N/P | Centromere |
| 45 | SSc | 23 | N/P | N/P | **973** | N/P | N/P | EJ |
| 46 | SSc | 53 | N/P | N/P | **3070** | N/P | N/P | None |
| 47 | SSc | N/P | 1:1280 | 316.0 | **4819** | N/P | N/P | None |

N/P=not provided. Exact IFA HEp-2 titer value not available for some samples, but all samples noted to be positive with clumpy nucleolar staining pattern (AC-9).
